# A Potential Role for mu-Opioids in Mediating the Positive Effects of Gratitude

**DOI:** 10.3389/fpsyg.2017.00868

**Published:** 2017-06-21

**Authors:** Max Henning, Glenn R. Fox, Jonas Kaplan, Hanna Damasio, Antonio Damasio

**Affiliations:** ^1^Psychology, Brain and Creativity Institute, University of Southern CaliforniaLos Angeles, CA, United States; ^2^Performance Science Institute, University of Southern CaliforniaLos Angeles, CA, United States

**Keywords:** gratitude, fMRI, stress-relief, social support, mu-opioid, health

## Abstract

Gratitude is a complex emotional feeling associated with universally desirable positive effects in personal, social, and physiological domains. Why or how gratitude achieves these functional outcomes is not clear. Toward the goal of identifying its' underlying physiological processes, we recently investigated the neural correlates of gratitude. In our study, participants were exposed to gratitude-inducing stimuli, and rated each according to how much gratitude it provoked. As expected, self-reported gratitude intensity correlated with brain activity in distinct regions of the medial pre-frontal cortex associated with social reward and moral cognition. Here we draw from our data and existing literature to offer a theoretical foundation for the physiological correlates of gratitude. We propose that mu-opioid signaling (1) accompanies the mental experience of gratitude, and (2) may account for the positive effects of gratitude on social relationships, subjective wellbeing, and physiological health.

## 1. Introduction

The intentional practice of gratitude brings our attention to the positive aspects of our lives, and empirical research indicates that this can lead to many powerful benefits (Emmons and McCullough, 2004). It is widely known that gratitude is beneficial, but a mechanistic understanding of why and how gratitude exerts these positive effects has remained elusive. A better understanding of the physiological processes underlying gratitude will offer a roadmap for the development of novel clinical interventions and personal strategies for living happier, healthier, more fulfilled lives, while also prompting us to rethink our approach to human interaction at all levels of society.

Definitions of gratitude typically describe a situational, social, and moral emotional feeling, a mediator of social cohesion, and a reinforcer of reciprocal prosocial behavior (McCullough et al., 2001). The most intense gratitude is provoked when we receive gifts or aid that fulfill a personal need, and especially when we understand that the aid was intentionally given for that purpose (Tesser et al., 1968; Lane and Anderson, 1976).

Previous studies have characterized significant positive effects of gratitude, but they have not closely examined the physiological mechanisms that underlie these effects. The purpose of the present discussion is to account for the positive effects of gratitude by identifying physiological mechanisms that are consistent with these effects, and whose involvement during the mental experience of gratitude is supported by behavioral and evolutionary considerations.

## 2. Neural correlates of gratitude

In 2015, we published a neuroimaging study that identified brain regions whose activation intensity correlated with the intensity of gratitude reported by our subjects. In designing our study, we turned to the USC Shoah Foundation's Visual History Archive, the world's largest repository of videotaped testimony from Holocaust survivors. A trend we observed among the testimonies was that survivors repeatedly reported receiving aid or gifts from strangers that often made the difference between life and death. The described “aid” ranged from sharing bread to being granted shelter by strangers at great personal risk during Nazi manhunts. We filtered the testimonies, flagging those that clearly described a benefactor, aid given, and a beneficiary, and transcribed the selected stories into second-person (e.g., “You are on the run from a Nazi manhunt; Your neighbors rescue you from the streets and help you into hiding for the winter, saving your life.”).

We created a database of 48 such stories from Holocaust survivors, describing receiving aid that ranged from relatively inconsequential to life-saving. Some aid met an acute need but did not require much effort on behalf of the benefactor (e.g., a cobbler giving away extra or damaged shoes) while other aid required tremendous benefactor effort, but was not useful to the beneficiary (e.g., being given peanuts that were procured at great risk but having an allergy that prevented eating them). Finally, some stories described life-saving aid that also put the benefactor in tremendous danger, such as sheltering a Jewish fugitive in ones' family home.

Research subjects were instructed to imagine themselves as the beneficiary in these stories, to feel the emotional context of the story as deeply as possible, and to imagine what it would feel like to receive the aid described. During this time, we collected functional Magnetic Resonance Imaging (fMRI) data, and after each story, participants were asked to rate how much gratitude they felt on a scale of 1–4. Their own ratings of gratitude were correlated in a general linear model with the BOLD signal activity during their reflection time. We hoped the results would identify core brain regions active during the mental experience of gratitude, and we predicted that these brain regions would be among those known to support moral reasoning and social cognition.

Indeed, a set of brain regions whose activity correlated with the intensity of reported gratitude was identified. These regions are located in the medial prefrontal cortex (mPFC), and include the peri-genual anterior cingulate cortex, and the more anterior portion of the mPFC. More generally, the event of receiving aid in our study elicited robust activity compared to baseline, implicating multiple overlapping social, stress-related, and somatosensory brain regions (O'Connell and Hofmann, 2011). This result aligned with our hypotheses, considering the regions identified have established roles in moral reasoning and social cognition (Zahn et al., 2008), and the involvement of these regions in gratitude has since been supported by a study from Kini et al. (2016).

Identifying neural correlates is an important step toward defining a physiological model of gratitude, and the results of our study have helped guide our thinking on the present topic. A lingering question from our study concerns what was being encoded by the brain activity we detected. One possibility is that because the brain activity was correlated with the intensity of self-reported gratitude for aid, the brain activity could represent the “value” of the aid to the beneficiary. In the context of our experiment, value for the beneficiary may be related to the reward or reduction in stress resulting from the aid received. This is relevant, considering plausible physiological correlates of gratitude must be consistent with the brain activity we identified, and must also be consistent with the well-characterized positive effects of gratitude on wellbeing and health.

## 3. The varied positive effects of gratitude

Gratitude quantifiably improves quality of life, with benefits that can be principally observed in social, personal, and health domains. Perhaps the most well-known effects of gratitude are **improvement in interpersonal relationships**, and increased prosocial behavior. Specifically, the reciprocal practice of gratitude has been shown to increase feelings of social inclusion and closeness in interpersonal relationships (Algoe et al., 2008, 2013), and individuals who feel grateful for a partner's help want to spend more time with them (Bartlett et al., 2011). Additionally, gratitude prompts major acts of reciprocal prosocial behavior (Ames et al., 2004) — an effect that has been attributed specifically to gratitude, and not to indebtedness or general positive affect alone (Bartlett and DeSteno, 2006). Indeed, the experimental induction of gratitude in subjects increases the likelihood of them “paying it forward” by sacrificing personal benefit to help others (Tsang, 2007). Finally, gratitude appears to be context-dependent, as the intensity of reported gratitude increases significantly when kindness exceeds our expectations (Wood et al., 2011). Hinting at physiological underpinnings, Sara Algoe's laboratory observed an increase in oxytocin release between romantic pair bonds after a gratitude intervention, although considering oxytocin has been ascribed a central role in mate selection and sexual attraction (Machin and Dunbar, 2011) the romantic nature of these relationships complicates the attribution of oxytocinergic processes to gratitude specifically (Algoe and Way, 2014).

KEY CONCEPT 1 **Gratitude is beneficial for social relationships**

Intentionally practiced or unexpectedly provoked, gratitude improves interpersonal relationships, increases feelings of social inclusion and closeness, predicts reciprocal prosocial behavior, and can reduce symptoms of mental illness.

In addition to its effects on social relationships and social behavior, gratitude has been shown to correlate with measures of subjective wellbeing and life-satisfaction. Teachers instructed to complete a gratitude intervention reported more meaning in their lives and lower levels of burnout (Chan, 2010), and another study identified gratitude as a better predictor of life-satisfaction than the big-five personality traits (Wood et al., 2008a). Corroborating this indirectly, the landmark Grant Study followed male Harvard sophomores for 70 years, searching for the life-history variables that predict life-satisfaction. The study concluded that warm social relationships throughout life have the greatest positive impact on life-satisfaction (Vaillant, 2012). Finally, in addition to the positive effects already mentioned, gratitude, and the close personal relationships it supports, have been associated with **improved physiological health** (Tops et al., 2013).

KEY CONCEPT 2 **Gratitude is beneficial for health**

Gratitude correlates with subjective wellbeing and improvements in physiological health. Specifically, gratitude is associated with increased life satisfaction, resiliency to health issues, and better sleep quality, in addition to lower levels of burnout, and reductions in stress, inflammation, and depression.

Studies of gratitude's effect on health report that subjects who score higher in assessments of dispositional propensity for gratitude are more resilient to health issues, cope better with heart failure, exhibit reduced levels of inflammatory biomarkers and depression, and also sleep better (Wood et al., 2009; Mills et al., 2015). These positive health effects appear to be mediated by gratitude specifically, as opposed to personality differences, as subjects participating in a gratitude-focused journaling condition reported fewer health problems and slept more hours per night (Emmons and McCullough, 2003).

It is apparent that developing a mindset of gratitude can significantly improve our quality of life, but an understanding of why and how these effects manifest requires a physiological model of gratitude. Unfortunately, the physiological correlates of gratitude have only been minimally studied in humans, which has limited our ability to understand the underlying mechanisms. However, certain stereotyped primate behaviors arise in social contexts and produce effects that are strikingly consistent with the social contexts and effects of gratitude observed in humans. For instance, it is clear that other species engage in reciprocal, cooperative, value-based behavioral exchanges, one example of which is social grooming, otherwise known as “allogrooming” (Emmons and McCullough, 2004). Primate allogrooming offers a compelling animal model of gratitude, and the physiological mechanisms underlying the effects of allogrooming in primates inform our search for the physiological mechanisms underlying the effects of gratitude in humans.

## 4. Allogrooming as an animal model of the physiological effects of gratitude

Allogrooming represents the intersection of sociality, reciprocal prosocial behavior, and improved health. Almost all social vertebrates engage in some form of allogrooming, but the dynamics of the behavior vary considerably between species. For example, impalas have been observed to engage in a tightly regulated, highly reciprocal allogrooming routine in which they take turns grooming the head and neck regions of their grooming partner (Schneeberger, 2016). This allogrooming behavior is relatively utilitarian, and serves the clear function of removing ectoparasites from regions that, if you were an impala, would be difficult to reach on your own. This utilitarian allogrooming dynamic contrasts with the socially oriented primate variety.

As with impalas and other mammals, primate allogrooming serves a hygienic function, but primates spend up to 17% of their waking hours grooming each other, far more than is necessary for hygienic reasons alone (Dunbar, 1991; Machin and Dunbar, 2011). The principle effect of primate allogrooming appears to be the maintenance of harmonious societies (Russell and Phelps, 2013); primate social groups tend to be larger than those of other animals, and their social relationships tend to be more complex, diverse, and long lasting (Machin and Dunbar, 2011). Allogrooming settles in-group tensions, maintains social bonds, reinforces social hierarchies, and provokes reciprocal prosocial behavior in the form of reciprocal grooming, food sharing, or coalitionary support (de Waal, 1997; Akinyi et al., 2013; Jaeggi and Gurven, 2013; Lutermann et al., 2013; Borgeaud and Bshary, 2015; Garrido et al., 2016; Richard et al., 2016). The parallels between the effects of allogrooming and the effects of gratitude on social behavior are apparent, and a possible link between the social effects of allogrooming and physiology emerges from the observation that when an individual is being groomed, they visibly relax and their heart rate decreases significantly (Grandi and Ishida, 2015).

This physiological effect of allogrooming has been attributed to C-Tactile (C-T) afferent sensory nerves that are selectively activated by body-temperature, stroking touch (Grandi, 2016; Olausson et al., 2016). C-T fibers have been widely implicated in social touch in primates as well as humans, and their activation increases hedonic liking of a person or place, manifesting in increased trust, compliance, and prosocial behavior (Björnsdotter et al., 2010). It has been theorized that the functional role of C-T fibers has shifted throughout mammalian evolution, from initially being related to parental nurturing in sub-primate species, to promoting social bonding in the more socially active primates (Olausson et al., 2010).

The relaxing effect of C-T fiber activation has been attributed to the release of oxytocin and endogenous mu-opioid peptides (Olausson et al., 2008). Our understanding of the molecular mediators of social behavior has long centered on oxytocin and vasopressin, but an alternative account offers an updated view. The Brain Opioid Theory of Social Attachment (BOTSA) suggests that while the oxytocin/vasopressin system is central to mate selection, attraction, and the maintenance of sub-primate social bonds, the oxytocin/vasopressin system cannot account for the unique dynamics of primate social behavior (Machin and Dunbar, 2011). Instead, the BOTSA contends that the complex, long-term, affective social relationships observed in primates and humans are principally mediated by endogenous mu-opioid peptide molecules and the associated mu-opioid receptor (MOR) system (Machin and Dunbar, 2011).

The MOR system has previously been implicated in mediating social reward (Trezza et al., 2011), and motivation (Kringelbach and Berridge, 2015), but the BOTSA posits a more central role for the MOR system in primate social behavior. The BOTSA contends that while oxytocin, vasopressin, dopamine, and serotonin systems may be implicated in the onset of primate and human bonding, as well as in intense romantic relationships, the MOR system is likely to be principally involved in the maintenance of the stable, long term relationships characteristic of our species (Machin and Dunbar, 2011). This posited role for the MOR system is supported by research demonstrating that primate solitication of allogrooming behavior is largely mediated by MOR interactions.

An early study discovered that talapoin monkeys treated with naloxone (general opioid antagonist) exhibited much higher rates of allogrooming than controls (Meller et al., 1980), and other studies demonstrated that (1) receipt of allogrooming in previously socially isolated monkeys increases the levels of MOR agonists in the central nervous system (CNS), and (2) solicitations for grooming were increased and decreased by administration of naloxone and morphine (a MOR agonist), respectively (Keverne et al., 1989; Martel et al., 1995). This suggests that opioid blockade reduced feelings of social connection in the monkeys and thus provoked the solicitation of grooming, while MOR activation increased feelings of social connection. This pattern is supported by a recent study of human subjects, in which Inagaki et al. demonstrated, with an experimental condition designed to evoke feelings of social connection, that inhibition of opioid interactions by naltrexone (general opioid antagonist) reduced feelings of social connection compared to controls (Inagaki et al., 2016).

The social effects of allogrooming are consistent with the social effects of gratitude, and theoretical and empirical evidence implicates the MOR system in mediating these effects in both allogrooming and gratitude. Implication of the MOR system in the social effects of gratitude offers a physiological process that may account for these effects; and in fact, the same MOR system may also account for the physiological health benefits that have been associated with gratitude.

## 5. CNS mu-opioids play a major role in physiological stress-reduction

In the context of biological systems, “health” effectively translates to “homeostasis.” “Homeostasis” describes a physiological state where the internal chemistries of the body are maintained within ranges conducive to relaxed and efficient continuation of the life process (Damasio and Carvalho, 2013). When deviations from these ranges occur, homeostasis deteriorates, and if left uncorrected, damage, disease, or death will result.

The “stress response” is a biochemical cascade provoked by homeostatic or environmental challenge that prepares the organism to confront an imminent threat to homeostasis. Acute stress has been shown to prepare an organism to fight or flee, but engaging a stress-response is energy intensive (Richard and Brian, 1989) and when the stress is not quickly resolved, homeostasis — and health — suffer. Indeed, chronic stress has been causally associated with deterioration in health and subjective wellbeing (Glaser and Kiecolt-Glaser, 2005; Rygula et al., 2005; Marin et al., 2011).

While the idea that the MOR system may be involved in the maintenance of long term social bonds is relatively new, their involvement in stress-reduction has been understood since their discovery in the 1970's. MOR agonists are released during conditions of physiological stress-relief (Curtis et al., 2001) as well as reward (Merrer et al., 2009) and they powerfully attenuate the stress-response (Wynne and Sarkar, 2013). Repeated stress reduction achieved through intentionally practicing gratitude could account for the positive health effects associated with gratitude.

Our 2015 study did not explicitly test whether physiological stress-reduction accompanied the mental experience of gratitude, however, some of the most intense gratitude reported by our subjects was (unsurprisingly) provoked by stories in which a stranger saved the protagonists' life at great personal risk. This alone is not sufficient grounds to claim involvement of the stress-reducing MOR system, but it is reasonable to expect that recognizing one's life is in danger produces stress; and it is similarly reasonable to expect that realizing that a stranger has gone out of their way to save your life would replace this stress with an intense feeling of gratitude.

Of course, not all gratitude-provoking situations involve stress-relief, but previous studies have noted that subjects report decreased levels of stress following the experimental induction of gratitude (Krause, 2006; Wood et al., 2008b, 2010). This indicates that even in gratitude-provoking conditions that do not involve stress-relief, MOR system activation appears likely, as subjective reports of decreased stress following the experimental induction of gratitude are **consistent with established effects of the MOR system**.

KEY CONCEPT 3 **Mu-opioids and stress-relief may account for the positive effects of gratitude**

The mu-opioid system is centrally involved in the restoration or improvement of homeostasis, as evidenced by its roles in analgesia, positive affect and reward, social motivation, long term affective bonds, and relaxation from stress. Meanwhile, the experience of gratitude is characterized by positive affect, reciprocal prosocial motivation, long term affective bonds, and subjective reports of stress reduction. Qualitative and behavioral parallels between the experience of gratitude and the effects of the mu-opioid system suggest mu-opioid involvement in the experience of gratitude, which could offer a physiological account of many of the positive effects of gratitude. Future studies are necessary to test this hypothesis.

## 6. Conclusions

The purpose of the current article has been to account for the positive effects of gratitude by exploring its underlying physiological correlates. To this end, we propose that large-scale activation of the MOR system during the mental experience of gratitude may account for the social, individual, and health effects of gratitude that have been observed. Specifically, we propose that the social effects of gratitude may be mediated largely by the MOR system in accordance with the Brain Opioid Theory of Social Attachment (Machin and Dunbar, 2011); and we propose that gratitude improves health by (1) reducing physiological stress, and (2) producing reward – both personal and social – in its place via the same MOR system. The effects of the MOR system on social relationships, individual wellbeing, and physiological health are well-supported, and consistent with the positive effects of gratitude we have described.

MORs are likely involved in all positive emotional experiences, and so implicating the MOR system as a principle mediator of the positive effects of gratitude could be taken to suggest that these positive effects may not be unique to gratitude. While it is likely that all positive mental experiences exert some degree of positive effects (see Barbara Frederikson's Broaden and Build Theory of Positive Emotions Fredrickson, 2004), these effects are likely to be magnified in gratitude. The feeling of gratitude integrates social and personal reward, and this combination likely produces a positive feedback loop that amplifies the positive effects beyond what could be achieved from a non-social rewarding experience.

We have made a case that activation of the MOR system may be a physiological correlate of gratitude, and may underlie its positive effects, but it is unlikely that opioidergic mechanisms act alone. Additional research will be necessary to tease apart the relative contributions made by the oxytocin, vasopressin, serotonin, dopamine, and other systems in producing the effects of this powerful social emotional feeling.

## 7. Implications

Physiological signals can modify qualitative aspects of mental experience, and mental experience can modify physiological state. In this frame, feelings can be understood as means to access body state, allowing the intentional practice of gratitude to reduce physiological stress and improve our health as part of the broader **concert of homeostatic processes**. And vice versa, the body can be a means to access the mind, allowing the direct reduction of physiological stress through massage or social touch to reduce mental stress, improving our emotional wellbeing. Elaboration of this research will help us further understand the systematic relationship between mind and body, informing the creation of novel strategies to live happier, more fulfilling, and healthier lives.

KEY CONCEPT 4 **Gratitude is best understood in a homeostatic context**

We propose that it is helpful to think of gratitude, and emotions more broadly, in a homeostatic context. Since gratitude is a positive emotion, it is reasonable to expect the conditions surrounding the experience of gratitude to be beneficial for homeostasis, while the opposite should be true for negative emotions. The recognition that subjective emotional experience is rooted in the homeostasis of our organism (1) facilitates basic research investigations of the physiological correlates of emotion, and (2) suggests that intentional mindfulness of our emotional state may improve behavioral decision-making.

By definition, the power of gratitude extends beyond the individual. A primary function of primate allogrooming is settling tensions and improving social group cohesion, and in human societies a similar effect is likely wrought through moral codes, religious systems, and philosophical appeals to humanity that advocate the intentional practice of gratitude. We nod to Seneca in contending that an empirically-driven resurgence of the intentional practice of gratitude could have significant positive implications for the harmony of the human civilization, from interpersonal relationships to international diplomacy.

## Author contributions

MH outlined and wrote the manuscript. GF wrote and edited the manuscript. JK, HD, and AD each provided writing, feedback and editing.

### Conflict of interest statement

The authors declare that the research was conducted in the absence of any commercial or financial relationships that could be construed as a potential conflict of interest.
